
JOURNAL INFORMATION
==============================
NLM Title Abbreviation: ChemistryOpen
Journal ID: ChemistryOpen
NLM Journal ID: 101594811
Journal ID: open

ChemistryOpen

EISSN: 2191-1363
Publisher: Wiley

ARTICLE INFORMATION
==============================
PMCID: PMC3775518
PMID: 24551553
DOI: 10.1002/open.201390018
Article version: 1
Subjects: News

Spotlights on our sister journals: ChemistryOpen 4/2013

Print publication date: 2013 Aug
Electronic publication date: 2013 Aug 26
Volume: 2
Issue: 4
First page: 132
Last page: 135
Copyright: Copyright © 2013 WILEY-VCH Verlag GmbH & Co. KGaA, Weinheim
Copyright year: 2013
License: Re-use of this article is permitted in accordance with the Creative Commons Deed, Attribution 2.5, which does not permit commercial exploitation.
License URL: https://creativecommons.org/licenses/by/2.5/

==============================
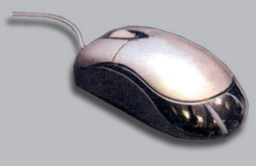

On these pages, we feature a selection of the excellent work that has recently been published in our sister journals. If you are reading these pages on a computer, click on any of the items to read the full article. Otherwise please see the DOIs for easy online access through Wiley Online Library.

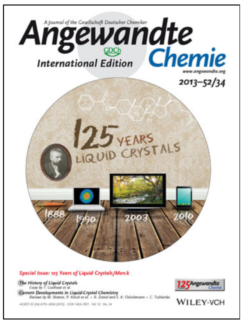

### Pathogen Detection

C. Anish, X. Guo, A. Wahlbrink, P. H. Seeberger*

### Plague Detection by Anti-carbohydrate Antibodies

**The sugar coat of "Black Death"** betrays it. The plague can be detected by monoclonal anti-carbohydrate antibodies. In a new technique, a plague specific oligosaccharide antigen (see structure) is synthesized. With the help of lipopolysaccharide (LPS)-specific monoclonal antibodies (mAbs) the presence of the plague pathogen *Yersinia pestis* can then be detected in serum from patients by using a glycan microarray.

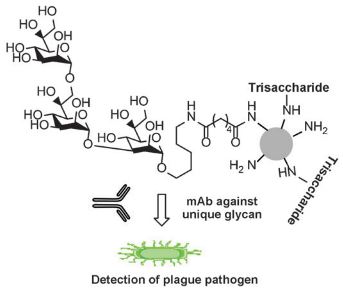

*Angew. Chem. Int. Ed.*
DOI: **10.1002/anie.201301633**

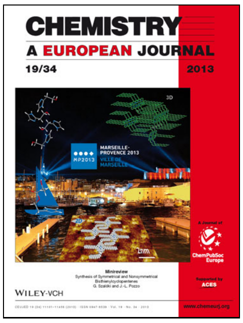

### Catalysis

M. Pérez-Lorenzo, B. Vaz, V. Salgueiriño, M. A. Correa-Duarte*

### Hollow-Shelled Nanoreactors Endowed with High Catalytic Activity

**Sleepy hollows wake up**: The design of different hollow-shelled nanoreactors is reviewed, analyzing the effects of both the shells and the active metal nanoparticles on the overall catalytic efficiency. Attention is focused on the methods used to prepare these hybrid nanocomposites and their different applications. Future perspectives on innovative architectures and further uses in catalysis are also addressed.

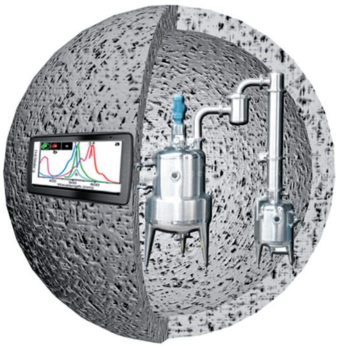

*Chem. Eur. J.*
DOI: **10.1002/chem.201301802**

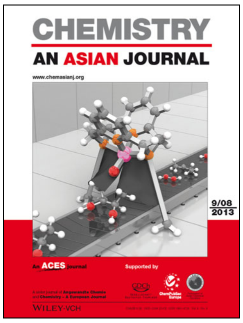

### Porphyrinoids

T. Higashino, A. Osuka*

### 2,3,17,18-Tetrahalohexaphyrins and the First Phlorin-type Hexaphyrins

**Exphlorin' all avenues**: 2,3,17,18-Tetrahalohexaphyrins were prepared by acid-catalyzed condensation of 3,4-dihalopyrroles and dipyrromethane-dicarbinol. These $\beta$-tetrahalogenated hexaphyrins display variable structural and electronic properties depending upon the halogen atom and the number of $\pi$-electrons. Tetrabromo- and tetrachloro[28]hexaphyrin were further reduced to provide phlorin-type hexaphyrins.

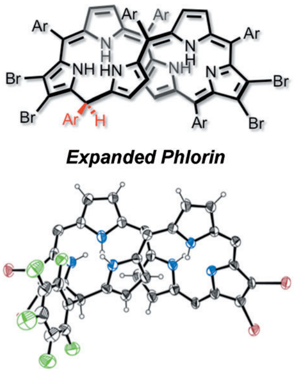

*Chem. Asian J.*
DOI: **10.1002/asia.201300474**

© 2013 Wiley-VCH Verlag GmbH & Co. KGaA, Weinheim

### Pheromones

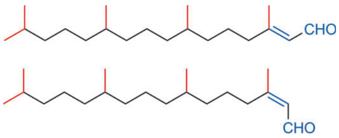

*ChemBioChem*
DOI: **10.1002/cbic.201300247**

*B. Fürstenau, L. Muñoz, M. Coca-Abia, G. Rosell, A. Guerrero,* C. Quero**

**Phytal: A Candidate Sex Pheromone Component of the Moroccan Locust *Dociostaurus maroccanus***

**Chemical communication in locusts**: A candidate sex pheromone component of the Moroccan locust *Dociostaurus maroccanus* has been identified by chromatographic, spectrometric and chiral HPLC analyses as phytal (*Z* and *E* isomers). The compound's biosynthesis proceeds from oxidation of phytol, and it displays attractant activity on both sexes.

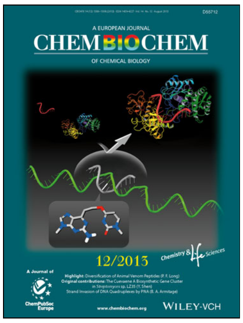

### Conducting polymers

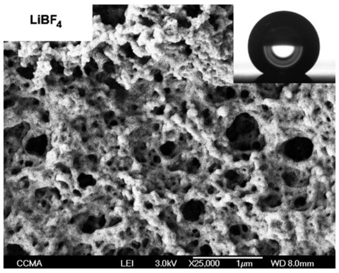

*ChemPhysChem*
DOI: **10.1002/cphc.201300448**

*O. Dunand, T. Darmanin, F. Guittard**

**Superhydrophobic Conducting Polymers Based on Hydrocarbon Poly(3,4-Ethylenedioxyselenophene)**

**Sitting on holes**: Superhydrophobic surfaces with high adhesion are obtained by electrodeposition of conducting polymers, hydrocarbon poly(3,4-ethylenedioxyselenophene). Such properties are specifically obtained using long alkyl chains ($C_{12}H_{25}$) and lithium tetrafluoroborate in propylene carbonate as electrolyte (see picture).

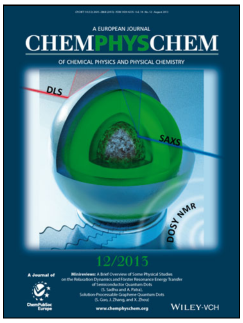

### Therapeutic Peptides

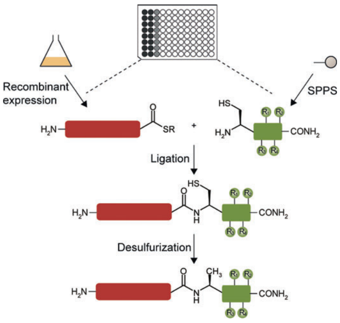

*ChemMedChem*
DOI: **10.1002/cmdc.201300290**

*L. Albertsen, S. Østergaard, J. F. Paulsson, J. C. Norrild,* K. Strømgaard**

**A Parallel Semisynthetic Approach for Structure–Activity Relationship Studies of Peptide YY**

**It's all parallel**: A semisynthetic approach was used to study the effect of C-terminal modifications of the appetite-reducing gut hormone peptide YY on interaction with its receptor. The approach involved ligation of a recombinant thioester building block with a suite of synthetic cysteine-containing peptides followed by desulfurization; this was performed in a parallel one-pot fashion.

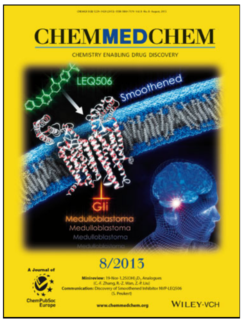

### Biomass Conversion

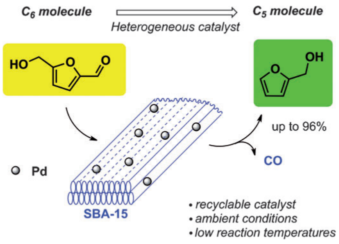

*ChemSusChem*
DOI: **10.1002/cssc.201300190**

*Y.-B. Huang, Z. Yang, M.-Y. Chen, J.-J. Dai, Q.-X. Guo, Y. Fu**

**Heterogeneous Palladium Catalysts for Decarbonylation of Biomass-Derived Molecules under Mild Conditions**

**Workin' 6 to 5**: A highly active heterogeneous catalyst, Pd/SBA-15(DP), promotes the selective decarbonylation of biomass-derived HMF to furfural alcohol with a high yield, up to 96%, in mild conditions. The route offers a simple and effective method of linking $C_6$ and $C_5$ molecules in the biomass conversion value chain. In addition to HMF the catalyst is effective towards the decarbonylation of other biomass-derived aldehydes, also.

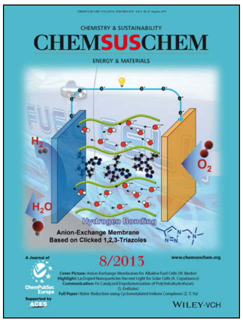

© 2013 Wiley-VCH Verlag GmbH & Co. KGaA, Weinheim

ChemPubSoc Europe

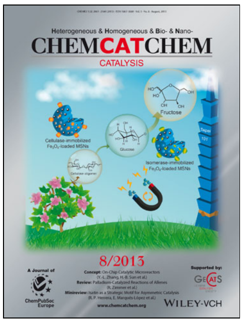

### Oligomerization

*A. N. Mlinar, O. C. Ho, G. G. Bong, A. T. Bell**

**The Effect of Noncatalytic Cations on the Activity and Selectivity of Nickel-Exchanged X Zeolites for Propene Oligomerization**

**Give me some space!** The presence of alkali metal and alkaline earth cations increases the available space within high aluminum content faujasite zeolites, which leads to higher propene oligomerization activity and increased branching in dimer products.

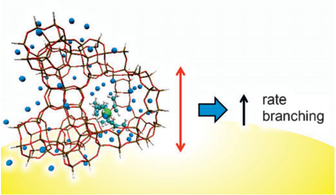

*ChemCatChem*
DOI: **10.1002/cctc.201300232**

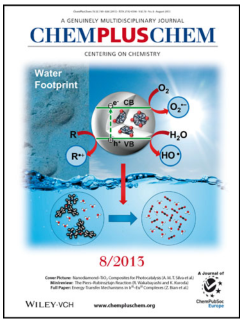

### Tubulins

*F. Calogero, S. Borrelli, G. Speciale, M. S. Christodoulou, D. Cartelli, D. Ballinari, F. Sola, C. Albanese, A. Ciavolella, D. Passarella,* G. Cappelletti, S. Pieraccini, M. Sironi**

**9-Fluorenone-2-Carboxylic Acid as a Scaffold for Tubulin Interacting Compounds**

**Tubulin as the target**: The introduction of a hydrophobic group at position 7 of 9-fluorenone-2-carboxylic acid (see structure) generates new tubulin binders, the design of which is suggested by modeling studies. The antiproliferative activity on two different cell lines, fluorescent microscopy, flow cytometry, and sedimentation assay tests confirmed the supposed mechanism.

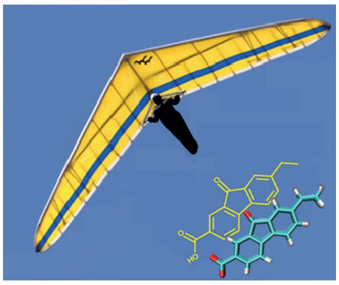

*ChemPlusChem*
DOI: **10.1002/cplu.201300036**

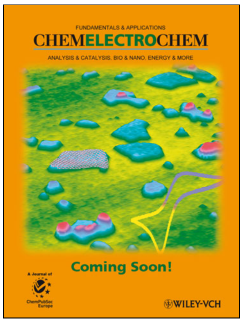

### Heterojunction Nanostructures

*X. Hou, X. Wang, B. Liu, Q. Wang, Z. Wang, D. Chen,* G. Shen**

**$SnO_2$@$TiO_2$ Heterojunction Nanostructures for Lithium-Ion Batteries and Self-Powered UV Photodetectors with Improved Performances**

**An effective strategy** is explored to prepare a hybrid material, which consists of rutile $SnO_2$ nanoparticles and rutile $TiO_2$ nanorods and can be effectively used in lithium-ion batteries and self-powered UV photodetectors.

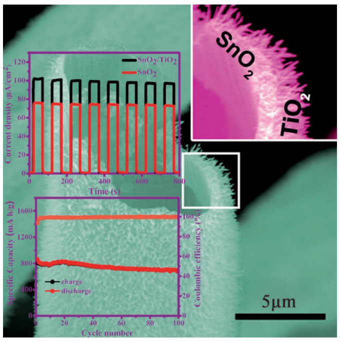

*ChemElectroChem*
DOI: **10.1002/celc.201300053**

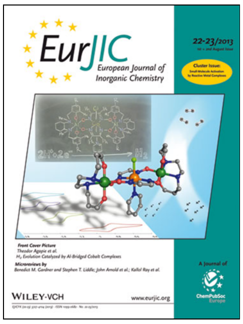

### 3D Spin-Crossover Systems

*D. Chiruta, J. Linares,* Y. Garcia,* P. R. Dahoo, M. Dimian*

**Analysis of the Hysteretic Behaviour of 3D Spin Crossover Compounds by Using an Ising-Like Model**

The effects of size, pressure and interactions on 3D spin crossover systems within the framework of an Ising-like model are discussed.

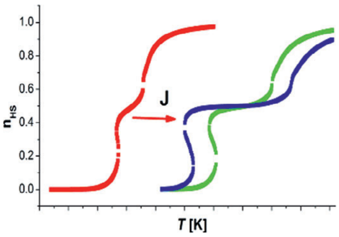

*Eur. J. Inorg. Chem.*
DOI: **10.1002/ejic.201300412**

### Lewis Base Catalysis

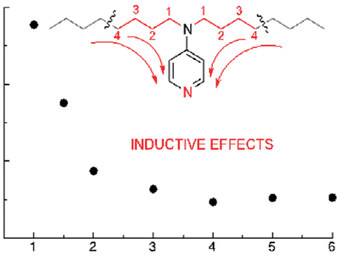

*Eur. J. Org. Chem.*
DOI: **10.1002/ejoc.201300486**

*R. Tandon, T. A. Nigst, H. Zipse**

**Inductive Effects through Alkyl Groups – How Long is Long Enough?**

Substituent effects of linear alkyl groups show a saturation effect beyond four carbon atoms for numerous properties of 4-(dialkylamino)pyridines as test substrates. These observations can be rationalized by using an increment-based method for the calculation of inductive substituent effects.

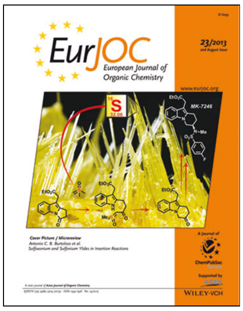

### N-Acylphthalimide Chemistry

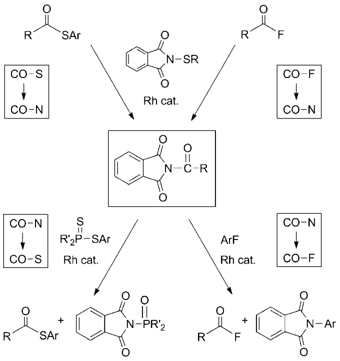

*Asian J. Org. Chem.*
DOI: **10.1002/ajoc.201300094**

*G. Li, M. Arisawa,* M. Yamaguchi**

**Rhodium-Catalyzed Synthesis and Reactions of *N*-Acylphthalimides**

**Method of many talents**: The synthesis and reactions of *N*-acylphthalimides are catalyzed by a complex of [RhH($PPh_3$)$_4$] and 1,2-bis(diphenylphosphino)benzene (dppBz). *N*-Acylphthalimides can be synthesized by the imidation of thioesters or acid fluorides with *N*-(organothio)phthalimides. Thioesters, *N*-(phosphinoyl)phthalimides, acid fluorides, and *N*-(tetrafluoroaryl)phthalimides can also all be synthesized by using this method.

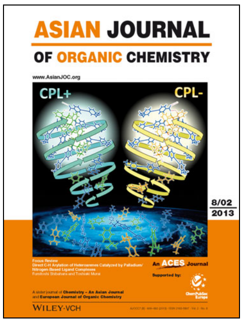

### Organic Synthesis

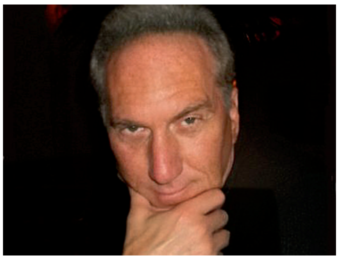

*ChemViews magazine*
DOI: **10.1002/chemv.201300087**

*Jonathan Rose*

**The Audience Wants More: A Good Article Tells a Story**

Professor Bruce Lipshutz, University of California, USA, discusses his continued fascination with organic chemistry and the new challenges brought by the need for organic chemists to avoid using environmentally harmful organic solvents. He also talks about how vital writing is in communicating science, and that with great science and good writing the passion behind the prose can be conveyed.

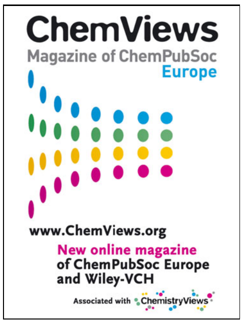

© 2013 Wiley-VCH Verlag GmbH & Co. KGaA, Weinheim
